# End stage renal disease as a modifier of the periodontal microbiome

**DOI:** 10.1186/s12882-015-0081-x

**Published:** 2015-06-09

**Authors:** Michel V. Furtado Araújo, Bo-Young Hong, Philip L. Fava, Shiza Khan, Joseph A. Burleson, George Fares, Wilner Samson, Linda D. Strausbaugh, Patricia I. Diaz, Effie Ioannidou

**Affiliations:** Division of Periodontology, Department of Oral Health and Diagnostic Sciences, School of Dental Medicine, University of Connecticut Health Center, 263 Farmington Ave, Farmington, CT 06030-1710 USA; Department of Community Medicine and Health Care, University of Connecticut Health Center, Farmington, CT USA; Bay State Medical Center, Springfield, MA USA; Division of Nephrology, Department of Medicine, School of Medicine, University of Connecticut Health Center, Farmington, CT USA; Center for Applied Genetics and Technologies, University of Connecticut, Storrs, CT USA

**Keywords:** Periodontitis, Periodontal microbiome, Chronic kidney disease, 16S rRNA amplicon sequencing, Dialysis vintage, End stage renal disease

## Abstract

**Background:**

Evidence supports high prevalence of periodontitis in patients with chronic kidney disease. Several renal factors have been proposed as possible modifiers of periodontitis pathogenesis in this population. In this cross sectional study, we investigated whether distinct microbial profiles in renal patients could explain high periodontitis prevalence.

**Methods:**

We characterized the subgingival microbiome in 14 End Stage Renal Disease (ESRD) and 13 control individuals with chronic periodontitis with similar demographic and clinical parameters. Medical, demographic and periodontal parameters were recorded. Subgingival biofilm samples were collected from the deepest pocket in two different quadrants and characterized via 454-pyrosequencing of the 16S rRNA gene.

**Results:**

We found 874 species-level operational taxonomic units (OTU) across samples. Renal and control groups did not differ in the individual proportions of periodontitis-associated taxa. However, in principal coordinate plots of distance among samples based on OTU prevalence, some renal patients clustered apart from controls, with the microbial communities of these outlier subjects showing less diversity. Univariate correlation analysis showed a significant negative correlation between dialysis vintage and community diversity.

**Conclusions:**

Within the study limitations, dialysis vintage was associated with a less diverse periodontal microbial community in ESRD suggesting the need for further research.

## Background

Chronic periodontitis, an infectious oral disease leading to tooth loss, is highly prevalent in Chronic Kidney Disease (CKD) with associations to malnutrition and inflammation [1–4]. Although recent epidemiological evidence has shown a periodontitis prevalence of 12.7 % in the general population [5], it increased up to ~39 % in some racial groups [6, 7] with a dose–response association in CKD [8]. Smaller studies in End Stage Renal Disease (ESRD) have reported a periodontitis prevalence of 29-64 % [9, 10]. Several renal disease-related factors have been hypothesized to contribute to the pathogenesis of periodontitis in CKD, including uremia and related immunosuppression and vitamin D deficiency [8, 11, 12]. Uremic toxins may potentially alter the oral ecosystem (hydrolysis of urea results in alkaline pH) promoting the growth of periodontal pathogens, in a similar manner to the demonstrated uremia-related changes in the gut environment [13, 14]. Studies using microbiological methods of limited scope have shown that ESRD individuals with periodontitis have increased levels of periodontal pathogens as compared to non-CKD controls [15]. However, with the recognition that chronic periodontitis is a complex infectious disease associated with polymicrobial biofilms, more emphasis is currently given to the study of subgingival bacteria in the context of a community [16]. It is thus unknown if the renal-related environmental effects of uremia contribute to oral community dysbiosis. Hence, the goal of this study was to compare the subgingival microbiomes of ESRD and non-CKD control individuals with chronic periodontitis using 454-pyrosequencing of the 16S rRNA gene, a technique that allows global profiling of microbial communities.

## Methods

### Study population

This cross-sectional pilot study included ESRD patients on hemodialysis as well as control, non-CKD, individuals. Patients with ESRD were recruited from the University of Connecticut Dialysis Center in Farmington, CT and the Springfield Dialysis Center in Springfield, MA. Control individuals were referred for periodontal treatment to the Periodontology Graduate Clinic at the University of Connecticut Health. All study procedures were approved by the Institutional Review Board of the University of Connecticut Health.

Participants were selected based on the following inclusion criteria: (1) at least one site with probing depth (PD) of 5 mm or more and two or more interproximal sites with clinical attachment loss (CAL) equal or more than 6 mm [17], (2) a minimum of 15 teeth, (3) no history of smoking, (4) no history of antibiotic use within the last month, and (5) no history of periodontal treatment within the last year. ESRD patients requiring by the nephrologist to take antibiotic prophylaxis prior to dental treatment were not excluded from this study since the single antibiotic dose was administered only 1 h prior to the sampling process and, therefore, did not affect the sampling process.

### Demographic and periodontal data collection

Study participants were informed about the study and signed the approved informed consent. Once eligibility for participation was established based on the inclusion/exclusion criteria, demographic data were recorded and medical history was reviewed. At this visit, participants received a comprehensive oral examination, which included number of missing teeth and plaque score (PS), defined as percentage of surfaces positive for plaque; pocket probing depths (PD), defined as the distance from the gingival margin to the base of the pocket measured; bleeding on probing (BoP), defined as percentage of surfaces positive for bleeding and clinical attachment levels (CAL), defined as the distance from the cemento-enamel junction to the base of the pocket measured. In order to assess the extent of periodontitis, the percentage of sites with PD ≥5 mm was calculated.

### Medical data

Medical history and biochemical data were extracted from the patient medical record. More specifically, most recent data on: 1) diabetes status, which was collected as a categorical variable, 2) dialysis vintage, defined by the number of dialysis years reflecting on ESRD chronicity [18], 3) serum albumin levels (g/dL), a biomarker of malnutrition and inflammation [19], 4) Kt/V (where K = dialyzer clearance of urea; t = dialysis time; and V = the distribution volume of urea) reflecting on dialysis adequacy and Blood Urea Nitrogen (BUN) (mg/dL), and 5) antibiotic prophylaxis in a regimen of amoxicillin 2 g or clindamycin 600 mg 1 h prior to the sampling visit.

### Biofilm sampling

Subgingival plaque samples were collected from the deepest pocket in two different quadrants after removal of supragingival plaque. The two plaque samples from the same individual were pooled and immediately placed in a polypropylene tube containing 50 μl Tris-EDTA (TE) buffer and stored at −80 °C.

### DNA isolation, 16S rRNA gene library preparation and sequencing

DNA was isolated from plaque samples using lysozyme and proteinase K treatment and a DNeasy Blood and Tissue kit (Qiagen) as previously described [20]. Positive controls (a known bacterial culture) and negative control samples for the assessment of sample contamination by foreign DNA (lysis and TE buffers without any sample) were also included.

Amplicon libraries of 16S rRNA gene V1-V2 hypervariable regions were generated in triplicate using fusion primers, which included universal primers 8 F 5′AGAGTTTGATCMTGGCTCAG3′ and 361R 5′CYIACTGCTGCCTCCCGTAG3′ [21], Roche Life Sciences’s 454 Lib-A adapters A and B and a unique multiplex identifier. PCR and library preparation procedures have been described previously [20]. Briefly, PCR reactions contained 10 ng of purified DNA, 1 U platinum *Taq* polymerase (Invitrogen), 1.5 mM MgCl_2_, 200 μM dNTPs, *Taq* buffer (1x), 0.5 μM of each forward and reverse primer and molecular grade water to a final volume of 25 μL. Thermal cycler conditions were: initial denaturation at 95 °C for 3 min; 25 cycles of denaturation at 95 °C for 30 s, annealing at 50 °C for 30 s and extension at 72 °C for 1 min; and a final extension step at 72 °C for 9 min. A negative PCR control with no added template was also included at this step. Combined triplicate amplicon libraries were sequenced in the forward direction using 454 Titanium chemistry on the 454-GS-FLX platform (454 Life Sciences). Sequences are available at the Short Reads Archive (SRP number pending).

### Sequence data processing

Sequence data were processed using a modification of the pipeline of Schloss *et al*. [22], as previously described [20], using mothur [23]. For Operational Taxonomic Unit (OTU)-based analysis, sequences were clustered using the average neighbor algorithm [24] and a 3 % dissimilarity cutoff. Individual sequences were classified using the ribosomal database project (RDP) classifier [25]. Template taxonomies included the RDP reference dataset and the Human Oral Microbiome Database (HOMD) [26]. OTUs were assigned a taxonomic identification based on the consensus assignment for the majority of sequences within each OTU. If a consensus taxonomy was not possible at the species level (based on HOMD), the nearest taxonomical rank at which a consensus was reached was reported. In such cases, the representative sequence from the OTU was also compared to the HOMD and if results showed more than 97 % similarity to an Oral Taxon (OT), the OT name of the top hit was reported in parentheses as part of the OTU taxonomy. Individually classified sequences were also grouped into phylotypes (from genus to phylum level) based on taxonomic identity.

Sequence libraries were sub-sampled to contain the same number of sequences to facilitate comparisons. Richness was evaluated by the number of observed OTUs and also by the number of estimated OTUs, as calculated with CatchAll [27], within a sample. Alpha-diversity was measured by the non-parametric Shannon index [28] and the inverse of the Simpson index [29]. β-diversity, which is a measure of differences among sample libraries, was determined with the Jaccard Index for comparison of communities based on membership (taxa prevalence) and the θ_YC_ distance [30] for comparison of communities according to their structure (taxa presence and relative proportions). Principal Coordinate Analysis (PCoA) of the distance among communities based on the Jaccard and θ_YC_ metrics was performed in mothur and graphs visualized using the rgl application within R (http://www.r-project.org/). Methods used for DNA isolation, amplicon library preparation, sequencing and microbial profile analysis have been previously validated using a mock community of oral microorganisms [20].

### Statistical analyses

Clinical and demographic data were compared via *t-*test, chi-square or Mann–Whitney tests for parametric and non-parametric data, as appropriate. Differences in α-diversity were evaluated by *t*-tests. Significant separation of clusters after PCoA was evaluated via Analysis of Molecular Variance (AMOVA) [31], as implemented in mothur. Differences in relative abundances of individual taxa were determined via LefSe [32], while differences in taxon prevalence were tested via chi-square. The false discovery rate method was used to adjust for multiple comparisons. To facilitate interpretation of prevalence data, we used the Chernoff bound to calculate the minimal relative abundance for which we could have 95 % certainty that we will observe at least one sequence at our sequencing effort [33]. Further, in CKD individuals, univariate Spearman’s correlation coefficient analysis was performed to assess the association between microbiome diversity and CKD-related variables with *P*-values < 0.05 considered to be statistically significant.

## Results

### Clinical and demographic descriptive analysis

Out of 52 ESRD and 17 control individuals, who gave consent to the study, 14 and 13 fulfilled the eligibility criteria, respectively. Among the 38 ESRD enrolled individuals, who failed the eligibility criteria, 6 patients were smokers, 14 patients had less than 15 teeth, 10 patients were periodontally healthy and 2 patients were on antibiotics for vascular access infections. In addition, four participants in the control group failed the eligibility criteria due to smoking. Table 1 shows the demographic and clinical characteristics of control and ESRD participants. Age, gender, ethnicity, diabetes status and PD, CAL, BoP and PS did not differ between groups. A marginal difference was found in the percentage of sites with PD ≥ 5 mm (*p* = 0.049), indicating greater periodontitis extent in the control group. The CAL and PD of sites sampled for microbiological analyses, however, were not different between groups (*p* = 0.74 and *p* = 0.06, respectively). Hence, the two groups were considered periodontally similar to test the research hypothesis.

**Table 1:** Clinical and demographic characteristics of Control and ESRD individuals

Characteristic	Control (n = 13)	ESRD (n = 14)	*p*-value
Age (years)^a^	48.4 ± 10.6	60.1 ± 16.1	0.55
Gender (% male) (n)^c^	69.2 (9)	57.1 (8)	0.52
Ethnicity (% non-whites) (n)^c^	61.5 (8)	42.9 (6)	0.56
Diabetes status (% yes) (n)^c^	15.4 (2)	50.0 (7)	0.06
Full mouth PD (mm)^b^	3.15 (2.4-5.0)	2.84 (2.4-4.3)	0.07
Full mouth CAL (mm)^b^	3.40 (2.4-6.0)	3.13 (2.5-7.5)	0.33
BoP (% of sites)^a^	52.0 ± 25.0	37.0 ± 25.0	0.13
PS (% of sites)^a^	59.0 ± 20.0	74.0 ± 25.0	0.10
PD ≥ 5 mm (% of sites)^b^	18.0 (4.0-65.0)	8.0 (3.0-45.0)	0.049
Sampled sites PD (mm)^a^	7.0 ± 0.8	6.3 ± 1.1	0.06
Sampled sites CAL (mm)^a^	7.6 ± 1.3	7.4 ± 2.1	0.74

Data represent mean ± standard deviation for normally distributed data, median (interquartile range) for non-normally distributed data, or frequencies (%) for dichotomous data

*PD*: pocket depth; *CAL*: clinical attachment level; *BoP* bleeding on probing; *PS* plaque score; *mm*: millimeters

Statistical Tests: Independent *t*-test (^a^); Mann Whitney (^b^); Chi-square test (^c^)

### Between group analysis of the subgingival microbiome

454-sequencing of 16S rRNA amplicon libraries from all individuals yielded 207,368 sequences after trimming and initial processing of sequence datasets. The range of sequences per library was 4,666-25,340. Libraries were normalized by random subsampling to contain the same number of reads (4,666 sequence reads per library). We found a total of 874 species-level (97 % similarity cutoff) OTUs among these normalized libraries, a number in agreement with recent taxonomic surveys of the subgingival microbiome.

First, we evaluated whether renal status influenced α-diversity, which is the diversity within a sample. The number of OTUs present per library in controls was 175.3 ± 45.4 as opposed to 147.1 ± 56.0 in the ESRD group. The inverse Simpson diversity index was 14.9 ± 8.1 in controls and 14.6 ± 11.0 in the ESRD group, while the non-parametric Shannon index measured 3.4 ± 0.6 in controls and 3.2 ± 1.0 in the ESRD group. None of these values were significantly different between groups.

**Fig. 1:**
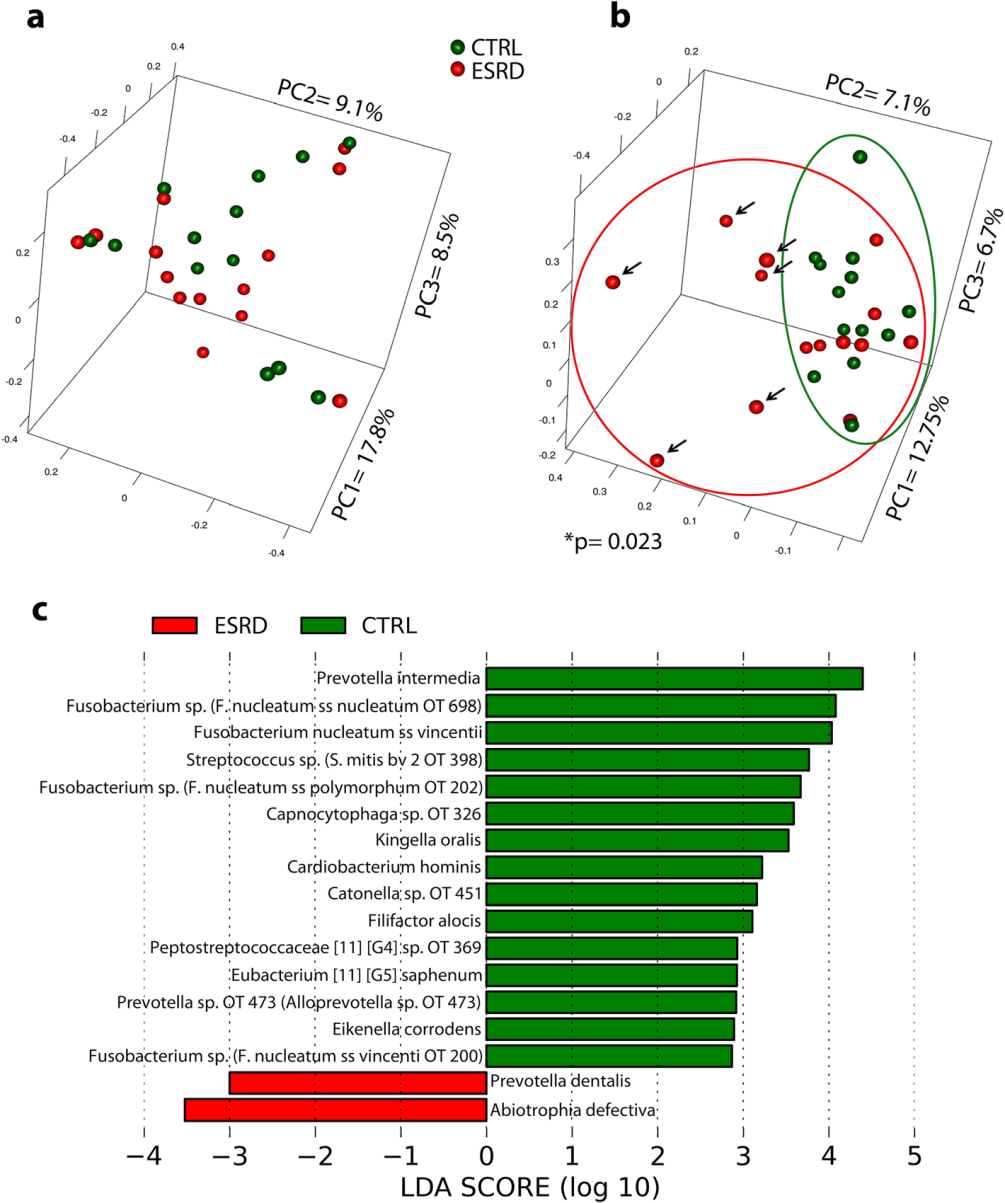
**a**: Difference in *β*-diversity in ESRD and Control samples. The plot was based on the θ_YC_ index, which measures distance among samples according to community structure. **b**: *β*-diversity comparisons (arrows indicate the ESRD-O samples). The plot was based on the Jaccard Index, which measures distance among samples according to taxa prevalence. **c**: Relative abundance of individual taxa. Individual OTUs with different relative proportion in ESRD (red bards) and Control groups (green bars). Differences in relative abundances were determined with LEfSe

Control and ESRD samples were then compared by PCoA analysis to evaluate the renal status effect on the global-scale composition of subgingival communities. Figure 1a shows a PCoA plot based on the θ_YC_ index, which measures distances among samples taking into account the presence and relative proportions of OTUs within communities. ESRD and control groups did not form discrete groups in this plot. However, when the distance among samples was calculated using the Jaccard index, which measures sample similarity based only on OTU prevalence, a significant difference was found (AMOVA = 0.023). Figure 1b shows the two data clouds in the Jaccard-based PCoA plot. As seen in this Figure, some ESRD samples clustered with the control group, while other ESRD samples were clearly apart (arrows in Fig. 1b). Analysis of sample clustering based on phylogenetic distance metrics yielded similar results (weighed UNIFRAC p > 0.05 and unweighted UNIFRAC *p* = 0.022). Therefore, these analyses indicated that renal status was not associated with a different subgingival community structure but could influence the composition of communities. It should nevertheless be noted that prevalence measures are dependent on depth of sampling. At the current sampling effort (4,666 sequences), an OTU should be present at a minimal relative abundance of 0.0017 % in order to have 95 % certainty that we will observe at least one sequence for this OTU [33].

Differences in the relative proportions and prevalence of individual OTUs between control and ESRD individuals were also evaluated. Figure 1c shows individual OTUs with a significantly different relative proportion between groups. This analysis indicates that only a small number of OTUs differed between groups, with most of these OTUs appearing as over-represented in the control group. Moreover, no differences in the prevalence of individual OTUs between groups were found (data not shown). These results suggested that renal status does not appear to be related to a specific subgingival microbial profile.

### Within-group analysis of the subgingival microbiome in CKD individuals

The heterogeneity observed in the ESRD group in terms of community composition (Fig. 1b) was further investigated by comparing the clinical and demographic participant characteristics in the two ESRD subgroups: a) ESRD-Control (ESRD-C) for their similarity with control group, and b) ESRD-outliers (ESRD-O) indicated by an arrow in Fig. 1b.

In a demographic comparison, the ESRD-O group was older (*p* = 0.07) with more males (*p* < 0.001) and more non-whites (p < 0.001) (Table 2) than the ESRD-C. The ESRD groups showed no differences in Kt/V and BUN (Table 1) reflecting dialysis adequacy. In terms of other medical confounders, the subgroups were similar in terms of diabetes status. When ESRD subgroups were compared in terms of dialysis vintage (*p* = 0.92) and albumin levels (*p* = 0.07), no statistically significant differences were found (Table 2). The subgroups were also compared in terms of antibiotic prophylaxis frequency, which could serve as an indicator of antibiotic exposure. We found that 67 % of the ESRD-O received prophylactic antibiotics in comparison to only 25 % in the ESRD-C group. This difference, however, did not reach a statistically significant level (*p* = 0.28).

**Table 2:** Comparison of clinical, demographic, alpha diversity, and CKD-related variables of ESRD-O and ESRD-C subgroups

Characteristic	ESRD-O (n = 6)	ESRD-C (n = 8)	*p*-value
	Demographic data		
Age (years)^a^	64.3 ± 13.4	55.8 ± 18.7	0.07
Gender (% male) (n)^c^	83.0 (5)	37.5 (3)	<0.001
Ethnicity (% non-whites) (n)^c^	67.0 (4)	25.0 (2)	<0.001
	Periodontal data		
PD (mm)^§^	3.0 ± 0.7	3.0 ± 0.5	0.50
CAL (mm)^a^	3.6 ± 1.0	3.8 ± 1.7	0.35
BoP (% of sites)^a^	40.0 ± 28.0	29.0 ± 20.0	0.40
PS (% of sites)^a^	73.0 ± 28.0	72.0 ± 28.0	0.89
PD ≥ 5 mm (% of sites)^b^	12.0 (5.0-45.0)	6.0 (3.0-36.0)	0.37
	Alpha diversity		
Number of observed OTUs^a^	101.8 ± 40.7	181.8 ± 39.0	0.004
Estimated OTUs^a^	169.0 ± 93.6	287.1 ± 68.3	0.029
Inverse simpson index^a^	8.1 ± 5.8	19.5 ± 11.7	0.036
NP-Shannon Index^a^	2.5 ± 1.2	3.7 ± 0.5	0.049
Shannon evenness^a^	0.5 ± 0.2	0.7 ± 0.1	1.00
	CKD-related variables		
Dialysis vintage (years)^b^	2.0 (1.25-4.0)	1 (1.0-5.0)	0.78
Antibiotic prophylaxis (% yes) (n)^c^	67.0 (4)	25.0 (2)	0.28
Albumin levels (g/dL)^b^	4.3 (3.7-4.4)	4.0 (3.0-4.3)	0.07
Diabetes status (% yes) (n)^c^	50.0 (3)	50.0 (4)	1.00
Dialysis adequacy (kt/V) ^b^	1.56 ± 0.18	1.85 ± 0.46	0.67
BUN (mg/dL)^a^	59.33 ± 13.04	55.05 ± 18.26	0.36

Data represent mean ± standard deviation for normally distributed data, median (interquartile range) for non-normally distributed data, or frequencies (%) for dichotomous data

*PD* pocket depth; *CAL* clinical attachment level; *BoP* bleeding on probing; *PS*: plaque score; *mm* millimeters; *OTUs*: Operational Taxonomic Units; g/dL: grams per deciliter; mg/dL: milligrams per deciliter

Statistical Tests: Independent *t*-test (^a^); Mann Whitney (^b^); Chi-square test (^c^)

Periodontal clinical parameters (mean PD, mean CAL, BoP, PS, and percentage of sites with PD ≥ 5 mm) did not show statistical differences in the ESRD subgroup analysis. However, comparisons of the subgingival microbiomes between ESRD subgroups showed that ESRD-O had decreased α-diversity compared to ESRD-C (Table 2), as well as compared to all control samples (data not shown). Therefore, the subgroups observed in Fig. 1b are likely to be due to a decreased prevalence of certain taxa in the ESRD-O. No specific taxa, however, could explain these differences when taxa prevalence between ESRD-C and ESRD-O were compared. This result was expected because of the broad separation among ESRD-O samples in PCoA plots, indicating they were all different from each other. Importantly, univariate Spearman’s correlation analysis in both groups revealed a statistically significant negative correlation between dialysis vintage and community diversity as measured by non-parametric Shannon index (r^2^ = −0.58, *p* = 0.04) and the inverse of the Simpson index (r^2^ = −0.71, *p* = 0.01) as well as community evenness as measured by the Shannon evenness index (r^2^ = −0.67, *p* = 0.02).

## Discussion

In an effort to understand the high prevalence of periodontitis in CKD, this pilot study examined the impact of ESRD on the periodontal microbiome using global microbial techniques. If we apply the polymicrobial synergy and dysbiosis model of periodontitis etiology [34] to the ESRD population, uremia may be the missing link perhaps causing the shift from microbial symbiosis to dysbiosis in the subgingival ecosystem. The “disrupted homeostatis” may trigger complicated host-microbial interactions [34], already compromised by functional abnormalities of neutrophils, monocytes/macrophages and dendritic cells [35–38] as well as impaired maturation of T helper cells [39].

The present pilot study observed no major differences in the subgingival microbiome between ESRD and control individuals, apart from decreased α-diversity in some ESRD individuals. Further, a small proportion of taxa with increased relative abundance were observed in the control group, including some periodontitis associated species such as *Prevotella intermedia* and *Filifactor alocis* (possibly explained by marginally higher periodontitis extent in the control group).

When groups were compared based on β-diversity metrics, composition disparities were identified between ESRD and control individuals confirming some heterogeneity and decreased diversity within the ESRD samples. Our findings are in agreement with investigations on the gut microbiome shown impressively similar clustering patterns with some ESRD individuals clustering tighter to the controls while others cluster apart [13]. Within the ESRD group, a significant negative correlation was found between dialysis vintage and microbial diversity and evenness indicating that the more the years on dialysis, not only the less the diverse but also the less even the microbial community was.

The human renal disease model is confounded by multiple factors including underlying diseases and therapeutic interventions. Among the therapeutic interventions, the dose, duration and frequency of antibiotic intake documented in intestinal communities could result in microbial shifts [40]. Assuming that our data on prophylactic antibiotics may reflect a pattern of frequent antibiotic administration, the decreased community diversity in some ESRD individuals could be explained and also confirmed by the inverse correlation of community diversity/evenness and dialysis vintage.

There has been limited evidence on the subgingival bacterial flora in CKD individuals using polymerase chain reaction (PCR) techniques with conflicting results and methodological bias [41] [15]. Although the present cross sectional design prevented any temporality assessment, the contemporary microbial methods strengthened the findings. Furthermore, this was a pilot study with small sample size, which limited result interpretation but validated our protocol hypothesis and supported feasibility. Although we have recognized the possible role of systemic antibiotics on microbial communities, data on antibiotic frequency and dose since dialysis initiation were not consistently available for this analysis.

Findings from this study showed that ESRD patients were adequately dialyzed as confirmed by the Kt/V measures. Therefore, in order to more accurately capture “the elephant of uremia” [42] and its “stamps” on the subgingival microbiome, our current conceptual model could be tested in advanced CKD stages prior to renal replacement therapy in a human CKD model for the etiopathogenesis of periodontitis in renal disease with controlled confounders such as chronic antibiotic usage. Future research in a larger population may be needed to examine the complete profile of these individuals at the level of the dysbiotic microbial community as well as the host response.

## Conclusions

Within the limitations of this pilot study, we did not find major differences in the subgingival microbiome between ESRD and control individuals, apart from decreased α-diversity in some ESRD individuals. However, the results showed a significant negative correlation between dialysis vintage and microbial diversity and evenness. This finding indicated that the more the years on dialysis, not only the less the diverse but also the less even the microbial community was.

## Abbreviations

ESRD: End stage renal disease
OUT: Operational taxonomic units
CKD: Chronic kidney disease
PS: Plaque score
PD: Pocket depth
BoP: Bleeding on probing
CAL: Clinical attachment levels
BUN: Blood urea nitrogen
RDP: Ribosomal database project
HOMD: Human oral microbiome database
OT: Oral taxon
PCoA: Principle coordinate analysis
AMOVA: Analysis of molecular variance
